# The effect of women’s body mass index on pelvic organ prolapse: a systematic review and meta analysis

**DOI:** 10.1186/s12978-021-01104-z

**Published:** 2021-02-19

**Authors:** Chernet Baye Zenebe, Wagaye Fentahun Chanie, Aster Berhe Aregawi, Tamiru Minwuye Andargie, Muhabaw Shumye Mihret

**Affiliations:** 1 Gynecology and Obstetrics Department, University of Gondar, Gondar, Ethiopia; 2 United Nations Population Fund Supported Maternal Health Project, College of Medicine and Health Sciences, University of Gondar, Gondar, Ethiopia; 3 United Nations Population Fund, Addis Ababa, Ethiopia; 4 Department of Clinical Midwifery, School of Midwifery, College of Medicine and Health Sciences, University of Gondar, Po. Box 196, Gondar, Ethiopia

**Keywords:** Body mass index, Meta-analysis, Obesity, Pelvic organ prolapse

## Abstract

**Background:**

Pelvic organ prolapse remains the public health challenge globally. Existing evidences report the effect of woman’s weight on the pelvic organ prolapse inconsistently and this urges the need of pooled body weight effect on the pelvic organ prolapse. Although there was a previous work on this regard, it included papers reported before June 18/2015. Thus, updated and comprehensive evidence in this aspect is essential to devise strategies for interventions.

**Objective:**

This review aimed at synthesizing evidence regarding the pooled effect of body weight on the pelvic organ prolapsed.

**Methods:**

For this review, we searched all available articles through databases including PubMed, Web of Sciences, CINAHL, JBI library, Cochran library, PsycInfo and EMBASE as well as grey literature including Mednar, worldwide science, PschEXTRA and Google scholar. We included cohort, case–control, cross-sectional and experimental studies which had been reported between March 30, 2005 to March 30, 2020. In the effect analysis, we utilized random model. The heterogeneity of the studies was determined by I2 statistic and the publication bias was checked by Egger’s regression test. Searching was limited to studies reported in the English language.

**Results:**

A total of 14 articles with 53,797 study participants were included in this systematic review (SR) and meta analysis (MA). The pooled result of this Meta analyses depict that body mass index (BMI) doesn’t have statistical significant association with pelvic organ prolapse.

**Conclusion:**

This review point out that women’s body mass index has no significant effect on the development of pelvic organ prolapse. However, the readers should interpret the result with cautions due to the presence of considerable limitations in this work.

*Trial registration* The protocol of this systematic review (SR) and meta analysis (MA) has been registered in PROSPERO databases with the Registration number of CRD42020186951

## Introduction

Pelvic organ prolapse (POP) is an anatomic support defect of the pelvic viscera. It may be resulted from a series of long term failure of supporting and suspension mechanisms of the uterus and the vaginal wall. The defect in the supporting structures results in downward displacement of structures that are normally located adjacent to the vaginal vault [1, 2].

Pelvic organ prolapsed (POP) severely affects women’s quality of life in several ways. Women with POP can feel different prolapse symptoms like “something coming down” and other urinary, bowel, and sexual symptoms [3–5]. It has socioeconomic and health consequences, affecting overall health and sexual function. It has been a major gynecologic problem in developed and developing nations [4, 6, 7].

Different risk factors such as increased maternal age and parity were identified to be linked to development of POP. However, most of those factors are non-modifiable. Similarly, maternal body mass index, which is a modifiable variable, also had been mentioned to be a determinant of POP although there are inconsistent reports across studies [8, 9].

As to the authors’ best knowledge, there is limited updated information on the pooled effect of maternal body mass index (BMI) on POP. In this regard, we obtained one systematic review (SR) and meta analyses (MA) regarding the effect of obesity on POP [10]. However, its search was limited on Pub Med/MEDLINE and included only papers published before June 18, 2015. We also found one related protocol which is limited to studies done in low and middle income countries. In addition, there is one review which is focused only on qualitative aspect in this aspect [11]

Thus, the current work was intended to fill these gaps. Accordingly, we included all the available published studies located in all accessible databases which had been reported between March 30/2005 to March 30/2020. By doing so, the current SR and MA come up with the evidences generated from existing studies. Thus, this review article was aimed at synthesizing the pooled effect of maternal BMI on pelvic organ prolapse globally.

## Method

### Protocol registration

The protocol of this SR and MA has been registered in the International Prospective Register of Systematic Reviews (PROSPERO) with the Registration number of CRD42020186951.

### Reporting

The Preferred Reporting Items for Systematic reviews and meta-analysis (PRISMA) guideline was utilized to report the results of this SR and MA.

### Databases and searching strategies

We searched through all available articles from PubMed using (((((("Women"[Mesh]) OR "Female"[Mesh]) AND ("Body Weight"[Mesh] OR "Weight Gain"[Mesh] OR "Body Weight Changes"[Mesh] OR "Weight Loss"[Mesh])) OR ("Obesity"[Mesh] OR "Obesity, Abdominal"[Mesh] OR "Obesity, Morbid"[Mesh] OR "Obesity, Maternal"[Mesh])) OR "Thinness"[Mesh]) AND "Pelvic Organ Prolapse"[Mesh]). We also tried to search using Web of Sciences, CINAHL, JBI library, Cochran library, PsycInfo and EMBASE databases though some of which are inaccessible. Similarly, we searched for grey literature through Mednar, worldwide science, PschEXTRA and Google scholar. In addition, we searched articles from the different institutional online research repositories and Reference lists of included studies using the following searching terms: “Body weight”, “Obesity”, “Pelvic organ prolapse”, “Body weight gain”, “POP”, “uterine prolapse”, “genital prolapse”, “enterocele”, “cystocele”, “anterior wall prolapse”,” rectocele” and “posterior wall prolapse” as a combination and as a single term.. We have conducted the search until March 30, 2020 and back to the previous recent 15 years.

### Inclusion and exclusion criteria

Articles included met the following criteria: (1) observational studies including experimental, cohort, case–control and cross-sectional, (2) published and unpublished studies which had been reported between March 30/2005 to March 30/2020, (3) studies contained the OR, RR or HR of BMI with respect to POP, and (4) Studies on POP and BMI reported in English.

However, conference papers, editorials, trials, reviews, program evaluations, and only qualitative studies and all studies which had reported only the mean effect (OR, RR or HR) of BMI with respect to POP were excluded since such results may bring about difficulty in aggregated OR interpretation (as the aggregated OR is intended to be interpreted and compared with the reference group (i.e., normal BMI)).

#### Outcome measurement

The outcome variable for this protocol is POP (Yes, No). All forms of prolapses reported as POP, uterine prolapse, genital prolapse, enterocele, cystocele/anterior wall prolapse or rectocele/posterior wall prolapse were counted as an outcome. Moreover, we included prolapses which had been either subjectively self-reported symptomatic prolapse or objectively measured prolapses as indicated by ICD codes, as well as prolapse measured through pelvic exams by trained professionals for all severities of prolapse. For the ease of data aggregation, reports of Baden–Walker Halfway grading system of grade 1 or more, or Pelvic Organ Prolapse Quantification (POP-Q) system stage I or more were considered comparable.

### Study selection and quality assessment

Primarily, all retrieved studies have been imported to Endnote version 7 citation managers. Consequently, duplicated studies were carefully removed from Endnote. Then, two independent authors screened and assessed the titles and abstracts and review the full texts. Any disagreement had been solved through discussion and communication with the primary authors of the studies. After the full text review, two investigators assessed the quality of the studies independently using the Joanna Brigg’s Institute (JBI) quality appraisal criteria adapted for respective study. Accordingly, studies with low risk i.e. whenever fitted to 50% and/or above quality assessment checklist criteria were included in this SR and MA.

### Data extraction

We extracted the first author of the study, year of publication, study area, design, study population, outcome variable measure, sample size, OR of BMI 30+, OR of BMI < 18.5 and OR of BMI (25.5–29.9).

We have focused on extracting of AOR as much as possible because of its importance for having adjusted and/or controlled possible confounders. For studies with no AOR, we have also searched for COR.

### Data analysis

A Stata version 11 statistical software was used for all statistical analysis. We used a random model for MA to estimate the pooled OR of BMI30+, OR of BMI < 18.5 and OR of BMI 25.5–29.9. We assessed the percentages of total variations across studies using I^2^ statistics. The values of I^2^, 25, 50, and 75% was represented low, moderate, and high heterogeneity respectively. Publication bias across studies was checked using Egger’s regression test.

## Results

### Findings and selection process

**Fig. 1:**
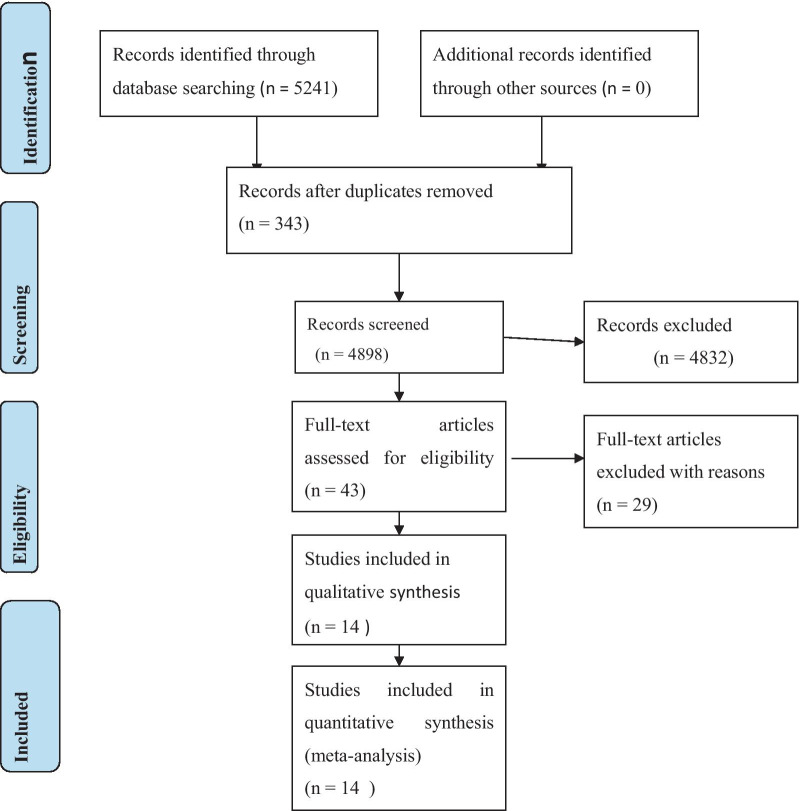
PRISMA flow diagram of selection process for the systematic review and meta analysis assessing the effect of BMI on POP

We obtained a total of 21,319 papers from all searching strategies. From these, we found about 5241 literature while we limit a searching filter date from March 30/2005 till March 30/2020. Upon filtration for duplication (n = 343), we selected 4898 articles. Thereafter, irrelevant studies (n = 4832) were removed based on the review of titles and abstracts. Among 43 articles which passed for further full-text review, about 29 articles were excluded for different reasons [3, 7–9, 12–36]. Finally, 14 articles were found relevant to assess the effect of BMI on POP (Fig. 1).

### Characteristics of the included studies

About 14 studies with 53,797 study participants were included in this SR and MA. Regarding the study area of the articles; two studies in Ethiopia [37, 38], one study in Tanzania [39],one study in United Arab Emirates [40], four studies in USA [41–44], three studies in Sweden [14, 45, 46], one study in UK [47], one study in New Zeeland [48], and one study in Nepal [49] were included. As far as the study design of the included articles concerned, we included seven studies case control [37–39, 43, 45, 46, 49], three cohort [14, 47, 48], two cross sectional [40, 44] and two RCT [41, 42] studies. Concerning the POP measurement in the included articles, seven studies measured POP objectively [37, 39, 41–43, 48, 49] and the remaining seven measured subjectively [14, 38, 40, 44–47] (Table 1).

**Table 1:** Characteristics of the include articles and their study participants’

Code	Authors	Year	Setting	Design	N	Study population	Measurement
1	Asresie et al	2016	Bahir Dar, Ethiopia	CC	370	Gynecologic patients (age > 18 years)	Stage 3 + Vs free (OM)
2	Elbiss et al	2015	United Arab Emirates	CC	429	All 30 + aged non-pregnant parous	Symptomatic (SM)
3	Henok A	2017	Southwest Ethiopia	CC	422	All > 15 years worked on firewood sales	Symptomatic (SM)
4	Masenga et al	2018	Kilimanjaro, Tanzania	CC	1047	Non-pregnant 18–90 year-age women	Stage 2 + Vs 0–1 (OM)
5	Kudish et al	2009	WSU, USA	RCT	16,608	Postmenopausal women with uteri aged 50 to 79	Stage l + (OM)
6	Tegerstedt et al	2005	Stockholm, Sweden	CC	859	All aged ≥ 15 years women	Symptomatic (SM)
7	Miedel et al	2009	Stockholm, Sweden	CC	859	All aged ≥ 15 years women	Symptomatic (SM)
8w	Kudish et al	2011	Washington DC, USA	RCT	11,185	Only white people	Stage ≥ II (OM)
8b	Kudish et al	2011	Washington DC, USA	RCT	800	Only black people	Stage ≥ II (OM)
8h	Kudish et al	2011	Washington DC, USA	RCT	665	Only Hipanic people	Stage ≥ II (OM)
9O	Whitecomb et al	2009	Kaiser, USA	CC	1137	Middle-aged and older women	Stage ≥ II (OM)
9S	Whitecomb et al	2009	Kaiser, USA	CC	2270	Middle-aged and older women	Symptomatic (SM)
9Oh	Whitecomb et al	2009	Kaiser, USA	CC	1137	Middle-aged and older women	≥ 0 cm (hymen and beyond)
10	Rortveit et al	2007	Northern California, USA	CS	2001	Age 40–69 and members of the KPMCPNC	Symptomatic (SM)
11	Dolan et al	2010	UK	Cohort	1782	Women who gave birth to their first child	Symptomatic (SM)
l2S	Glazener et al	2012	UK and New Zealand	Cohort	3763	Women gave birth 12 years back	Symptomatic (SM)
12OJ	Glazener et al	2012	UK and New Zealand	Cohort	762	Women gave birth 12 years back	≥ 0 cm (hymen and beyond)
13	Bohlin et al	2017	Sweden	Cohort	7209	Women at 1 year after primary POP surgery	Symptomatic (SM)
14	Devkota et al	2019	Kaski district, Nepal	CC	492	Non-pregnant 18–60 aged, with no hysterectomy	Stage ≥ I

### The effect of BMI on POP

**Fig. 2:**
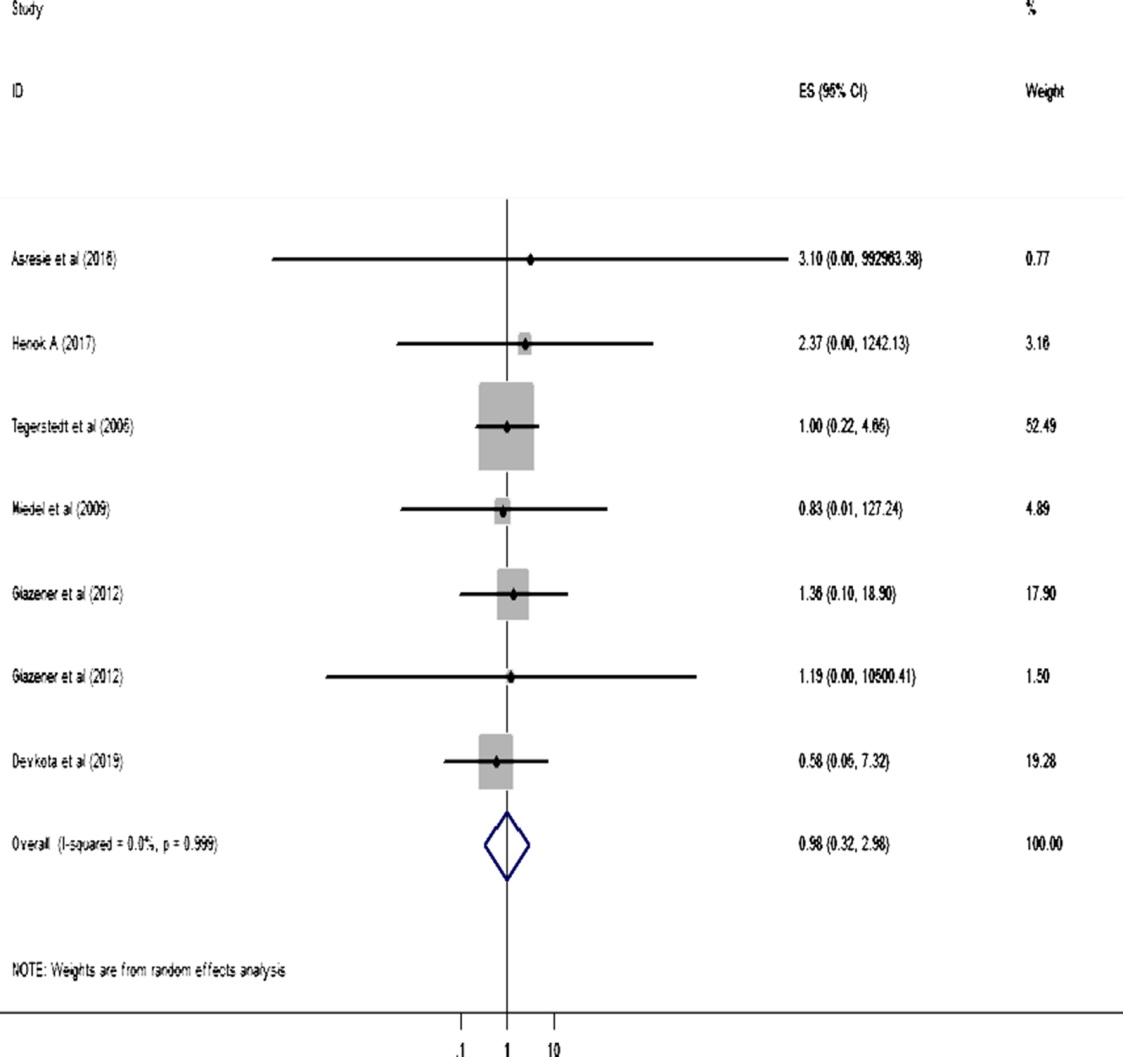
Forest plot showing the effect of BMI < 18.5 kg/m^2^ on pelvic organ prolapse

**Fig. 3:**
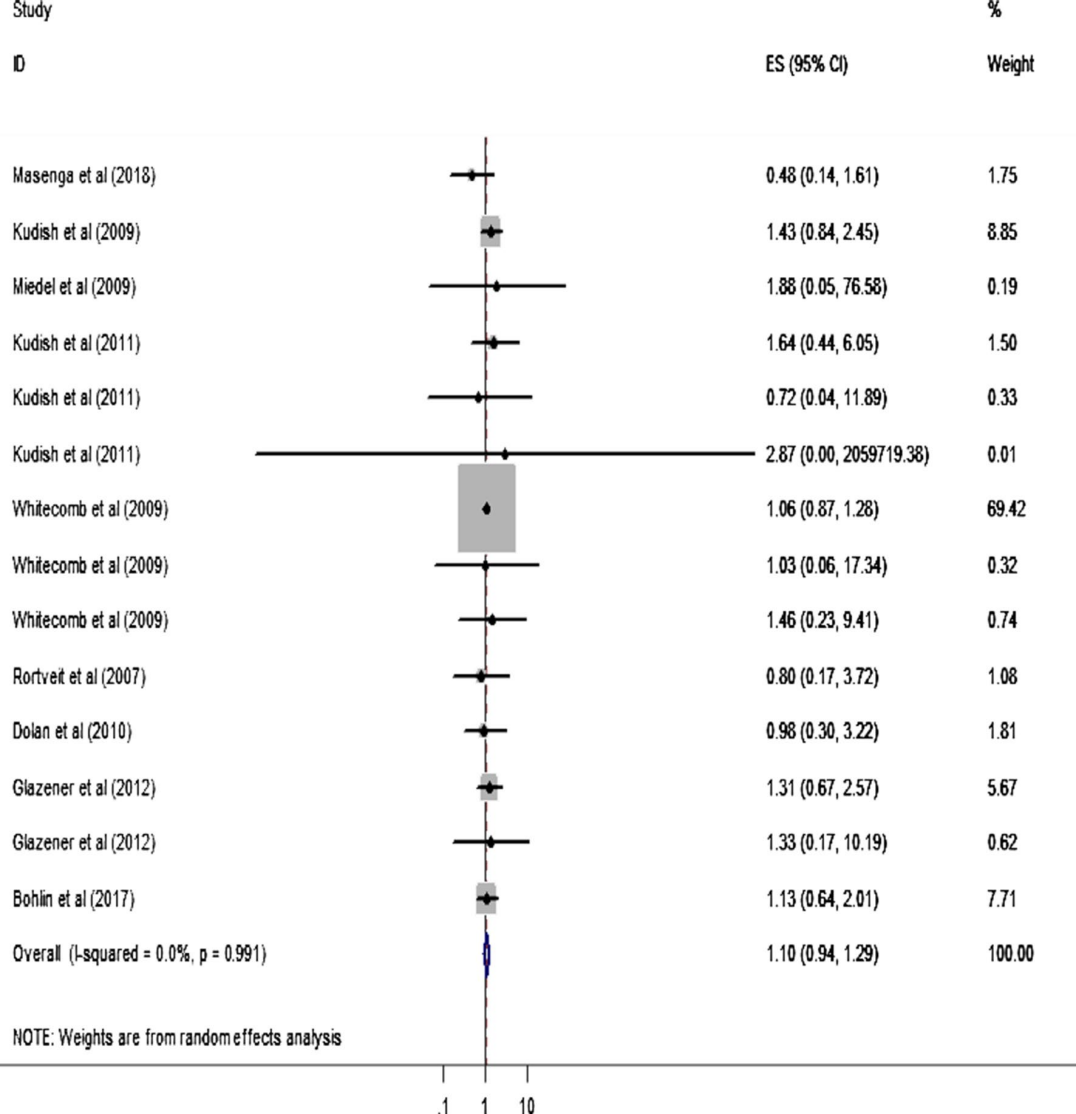
Forest plot showing the effect of BMI (25.5–29.9) kg/m^2^ on pelvic organ prolapse

**Fig. 4:**
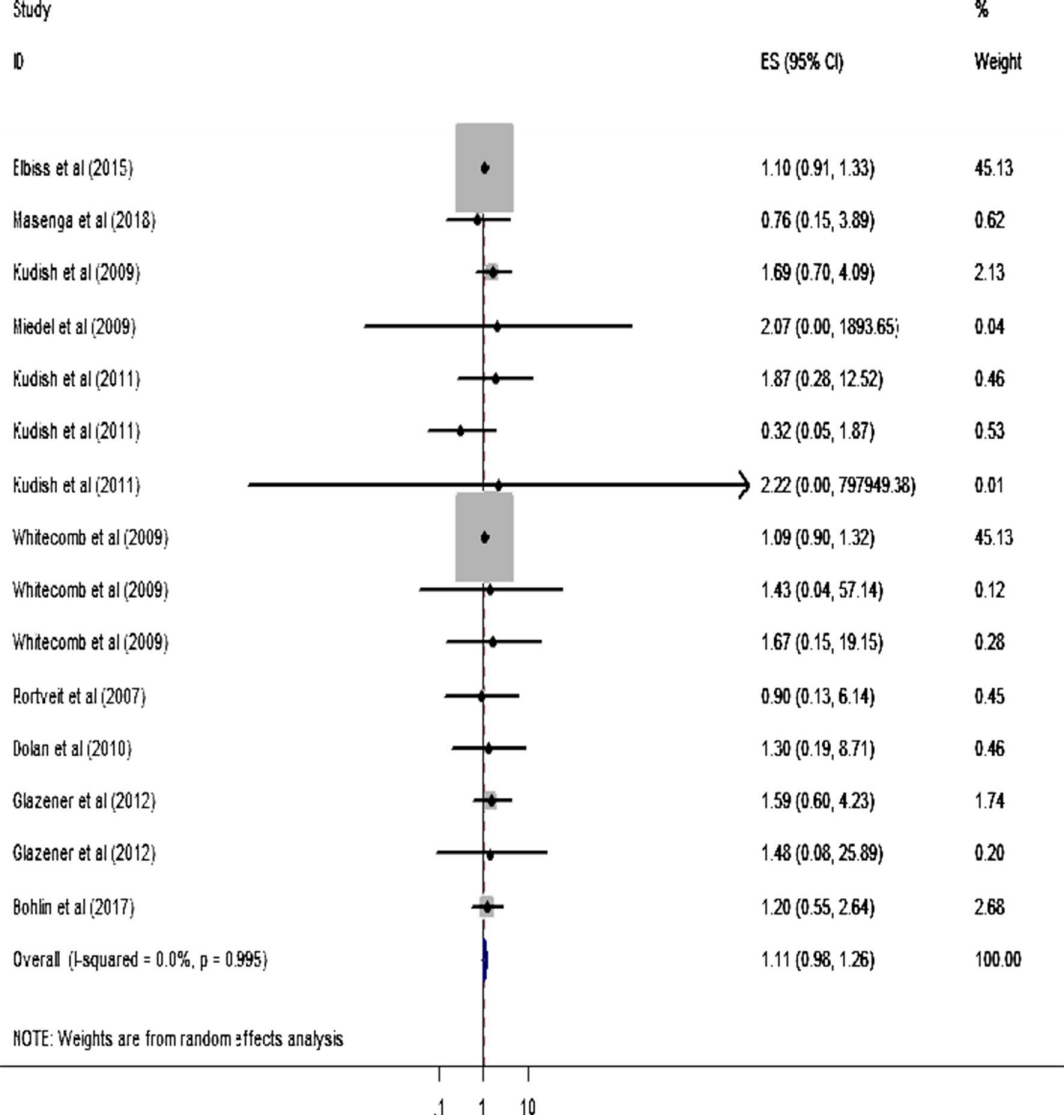
Forest plot showing the effect of BMI ≥ 30 kg/m^2^ on pelvic organ prolapse

Two studies reported the statistical significant association between BMI < 18.5 kg/m^2^ and POP [37, 38]. Likewise, five studies [41–43, 45, 48] reported that BMI of 25–29.9 kg/m^2^ had significant association with POP. Similarly, five studies [14, 41–43, 48] presented the finding exhibiting the significant association between BMI ≥ 30 kg/m^2^ and POP. In the MA, however, no statistical significant association is observed between each category of BMI and POP for all included articles. Similarly, the MA results depict that the pooled (overall) effect of each category of BMI on POP is statistically insignificant (Figs. 2, 3 and 4).

## Discussion

This SR and MA aimed at synthesizing the pooled effect of BMI on POP occurrences. The pooled MA results indicate that BMI index has no significant association with POP. This contradicts the result of previous similar work [10]. The previous SR and MA work was included papers published June 18, 2015. On the other hand, the current work included articles published between March 30/2005 and March 30/2020. Therefore, one possible justification for the discrepancy on the effect of BMI on POP could be publication time difference across included papers. In this regard, the former study is differed from the current study on two perspectives. First, the former study had included all eligible studies published from June 18/2015 backward unlike the current study which has not included articles published March 30/2005 back. Second, the previous MA and SR study had not considered the recent studies published since June 19/2015 onwards in contrast to the current study which included papers published till March 30/2020. Over time trend, the life styles of people are continually changing, and BMI is tremendously sensitive to changes in life styles.

The findings of this study should be interpreted with cautions as the study has a number of limitations. First of all, there is a high heterogeneity in definition of POP and categories of BMI across included studies. In this aspect, certain studies had measured POP subjectively (symptomatic based) [38, 45, 46] while others reported objectively [37, 48, 49]. There were also variations in cut-off points in POP definitions even within studies reported objectively ranged from stage ≥ 1[49] to stage ≥ 3 [37]. Second, some studies reported BMI’s category exhaustively (< 18.5 kg/m^2^, 18.5–25.5 kg/m^2^, 25.5–29.9 kg/m^2^ and ≥ 30 kg/m^2^) while others reported it in a simple way (< 18.5 kg/m^2^, 18.5–25.5 kg/m^2^ and ≥ 25 kg/m^2^). Even other else reported a single figure of the mean value of BMI. Third, some of the studies presented only its crude odds ratio unlike the rests which had included the adjusted odds ratio too. Lastly, there was a quite disparity in study participants across included studies (Fig. 3). Therefore, a non- significant association between BMI and POP in the pooled result could be attributed to the aforesaid shortcomings of this study.

## Conclusion

In this SR and MA, BMI has no pooled significant association with POP. However, the readers should interpret the result with cautions due to the presence of considerable limitations in this work.

## Limitations

As the categories of BMI had been reported inconsistently across literature, we forced to report the findings of these variables with some sort of variation in categories aggregately. In addition, this SR and MA might miss important related articles as some important studies had reported the findings in different way which made the data extraction difficult and the data interpretation hard.

## Data Availability

The datasets employed in the current study can be available from the corresponding author upon the reasonable request.

## Abbreviations

AOR: Adjusted odds ratio
BMI: Body mass index
COR: Crude odds ratio
MA: Meta analyses
POP: Pelvic organ prolapse
PROSPERO: Prospective Register of Systematic Reviews
SR: Systematic reviews
UNFPA: United Nation

## References

[1] Hallock JL, Handa VL (2016). The epidemiology of pelvic floor disorders and childbirth: an update. Obstet GynecolClin N Am.

[2] Merrell J, Brethauer S, Windover A, Ashton K, Heinberg L (2012). Psychosocial correlates of pelvic floor disorders in women seeking bariatric surgery. SurgObesRelat Dis.

[3] Myers DL, Sung VW, Richter HE, Creasman J, Subak LL (2012). Prolapse symptoms in overweight and obese women before and after weight loss. Female Pelvic Med ReconstrSurg.

[4] Ramalingam K, Monga A (2015). Obesity and pelvic floor dysfunction. Best Pract Res Clin Obstet Gynaecol.

[5] What to do about pelvic organ prolapse. Many women are living with this uncomfortable condition. Here are your next steps if you're one of them. Harvard women's health watch. 2014;21(10):4. 25178133

[6] Bilgic D, Gokyildiz S, KizilkayaBeji N, Yalcin O, Gungor UF (2019). Quality of life and sexual function in obese women with pelvic floor dysfunction. Women Health.

[7] Rodríguez-Mias NL, Martínez-Franco E, Aguado J, Sánchez E, Amat-Tardiu L (2015). Pelvic organ prolapse and stress urinary incontinence, do they share the same risk factors?. Eur J Obstet GynecolReprod Biol.

[8] Washington BB, Erekson EA, Kassis NC, Myers DL (2010). The association between obesity and stage II or greater prolapse. Am J Obstet Gynecol.

[9] Awwad J, Sayegh R, Yeretzian J, Deeb ME (2012). Prevalence, risk factors, and predictors of pelvic organ prolapse: a community-based study. Menopause (New York, NY).

[10] Giri A, Hartmann KE, Hellwege JN, Velez Edwards DR, Edwards TL (2017). Obesity and pelvic organ prolapse: a systematic review and meta-analysis of observational studies. Am J Obstet Gynecol.

[11] Thubert T, Deffieux X, Letouzey V, Hermieu JF (2012). Obesity and urogynecology: a systematic review. Progres en urologie: journal de l'Associationfrancaised'urologieet de la Societefrancaised'urologie.

[12] Akmel M, Segni H (2012). Pelvic organ prolapse in Jimma University specialized hospital, southwest ethiopia. Ethiop J Health Sci.

[13] Bodner-Adler B, Kimberger O, Laml T, Halpern K, Beitl C, Umek W (2019). Prevalence and risk factors for pelvic floor disorders during early and late pregnancy in a cohort of Austrian women. Arch Gynecol Obstet.

[14] Bohlin KS, Ankardal M, Nüssler E, Lindkvist H, Milsom I (2018). Factors influencing the outcome of surgery for pelvic organ prolapse. Int Urogynecol J.

[15] Chen HY, Chung YW, Lin WY, Wang JC, Tsai FJ, Tsai CH (2008). Collagen type 3 alpha 1 polymorphism and risk of pelvic organ prolapse. Int J Gynaecol Obstet.

[16] Chung SH, Kim WB (2018). Various approaches and treatments for pelvic organ prolapse in women. J Menopausal Med.

[17] Eleje G, Udegbunam O, Ofojebe C, Adichie C (2014). Determinants and management outcomes of pelvic organ prolapse in a low resource setting. Ann Med Health Sci Res.

[18] Gray T, Money-Taylor J, Li W, Farkas AG, Campbell PC, Radley SC (2019). What is the effect of body mass index on subjective outcome following vaginal hysterectomy for prolapse?. Int Neurourol J.

[19] Li Z, Xu T, Li Z, Gong J, Liu Q, Wang Y (2018). An epidemiologic study on symptomatic pelvic organ prolapse in obese Chinese women: a population-based study in China. Diabetes MetabSyndrObes Targets Ther.

[20] Milsom I, Gyhagen M (2019). Breaking news in the prediction of pelvic floor disorders. Best Pract Res Clin Obstet Gynaecol.

[21] Nakad B, Fares F, Azzam N, Feiner B, Zilberlicht A, Abramov Y (2017). Estrogen receptor and laminin genetic polymorphism among women with pelvic organ prolapse. Taiwan J Obstet Gynecol.

[22] Quiroz LH, Muñoz A, Shippey SH, Gutman RE, Handa VL (2010). Vaginal parity and pelvic organ prolapse. J Reprod Med.

[23] Shalom DF, Lin SN, St Louis S, Winkler HA (2012). Effect of age, body mass index, and parity on Pelvic Organ Prolapse Quantification system measurements in women with symptomatic pelvic organ prolapse. J Obstet Gynaecol Res.

[24] Smith FJ, Holman CD, Moorin RE, Tsokos N (2010). Lifetime risk of undergoing surgery for pelvic organ prolapse. Obstet Gynecol.

[25] Swift SE, Pound T, Dias JK (2001). Case-control study of etiologic factors in the development of severe pelvic organ prolapse. Int Urogynecol J Pelvic Floor Dysfunct.

[26] Thapa S, Angdembe M, Chauhan D, Joshi R (2014). Determinants of pelvic organ prolapse among the women of the western part of Nepal: a case-control study. J Obstet Gynaecol Res.

[27] Wusu-Ansah OK, Opare-Addo HS (2008). Pelvic organ prolapse in rural Ghana. Int J Gynaecol Obstet.

[28] Braekken IH, Majida M, EllströmEngh M, Holme IM, Bø K (2009). Pelvic floor function is independently associated with pelvic organ prolapse. BJOG Int J Obstet Gynaecol.

[29] Chen Y, Johnson B, Li F, King WC, Connell KA, Guess MK (2016). The effect of body mass index on pelvic floor support 1 year postpartum. Reprod Sci (Thousand Oaks, Calif).

[30] Diez-Itza I, Arrue M, Ibañez L, Paredes J, Murgiondo A, Sarasqueta C (2011). Influence of mode of delivery on pelvic organ support 6 months postpartum. Gynecol Obstet Invest.

[31] Forsman M, Iliadou A, Magnusson P, Falconer C, Altman D (2008). Diabetes and obesity-related risks for pelvic reconstructive surgery in a cohort of Swedish twins. Diabetes Care.

[32] Fritel X, Varnoux N, Zins M, Breart G, Ringa V (2009). Symptomatic pelvic organ prolapse at midlife, quality of life, and risk factors. Obstet Gynecol.

[33] Gyhagen M, Bullarbo M, Nielsen TF, Milsom I (2013). Prevalence and risk factors for pelvic organ prolapse 20 years after childbirth: a national cohort study in singleton primiparae after vaginal or caesarean delivery. BJOG Int J Obstet Gynaecol.

[34] Isık H, Aynıoglu O, Sahbaz A, Selimoglu R, Timur H, Harma M (2016). Are hypertension and diabetes mellitus risk factors for pelvic organ prolapse?. Eur J Obstet GynecolReprod Biol.

[35] Rodrigues AM, Girão MJ, da Silva ID, Sartori MG, Martins Kde F, Castro RA (2008). COL1A1Sp1-binding site polymorphism as a risk factor for genital prolapse. Int Urogynecol J Pelvic Floor Dysfunct.

[36] Slieker-ten Hove MC, Pool-Goudzwaard AL, Eijkemans MJ, Steegers-Theunissen RP, Burger CW, Vierhout ME (2009). Symptomatic pelvic organ prolapse and possible risk factors in a general population. Am J Obstet Gynecol.

[37] Asresie A, Admassu E, Setegn T (2016). Determinants of pelvic organ prolapse among gynecologic patients in Bahir Dar, North West Ethiopia: a case–control study. Int J Women's Health.

[38] Henok A (2017). Prevalence and factors associated with pelvic organ prolapse among pedestrian back-loading women in bench Maji Zone. Ethiop J Health Sci.

[39] Masenga GG, Shayo BC, Rasch V (2018). Prevalence and risk factors for pelvic organ prolapse in Kilimanjaro, Tanzania: a population based study in Tanzanian rural community. PLoS ONE.

[40] Elbiss HM, Osman N, Hammad FT (2015). Prevalence, risk factors and severity of symptoms of pelvic organ prolapse among Emirati women. BMC Urol.

[41] Kudish BI, Iglesia CB, Sokol RJ, Cochrane B, Richter HE, Larson J (2009). Effect of weight change on natural history of pelvic organ prolapse. Obstet Gynecol.

[42] Kudish BI, Iglesia CB, Gutman RE, Sokol AI, Rodgers AK, Gass M (2011). Risk factors for prolapse development in white, black, and Hispanic women. Female Pelvic Med ReconstrSurg.

[43] Whitcomb EL, Rortveit G, Brown JS, Creasman JM, Thom DH, Van Den Eeden SK (2009). Racial differences in pelvic organ prolapse. Obstet Gynecol.

[44] Rortveit G, Brown JS, Thom DH, Van Den Eeden SK, Creasman JM, Subak LL (2007). Symptomatic pelvic organ prolapse: prevalence and risk factors in a population-based, racially diverse cohort. Obstet Gynecol.

[45] Miedel A, Tegerstedt G, Maehle-Schmidt M, Nyrén O, Hammarström M (2009). Nonobstetric risk factors for symptomatic pelvic organ prolapse. Obstet Gynecol.

[46] Tegerstedt G, Miedel A, Maehle-Schmidt M, Nyrén O, Hammarström M (2006). Obstetric risk factors for symptomatic prolapse: a population-based approach. Am J Obstet Gynecol.

[47] Dolan LM, Hilton P (2010). Obstetric risk factors and pelvic floor dysfunction 20 years after first delivery. Int Urogynecol J.

[48] Glazener C, Elders A, MacArthur C, Lancashire RJ, Herbison P, Hagen S (2013). Childbirth and prolapse: long-term associations with the symptoms and objective measurement of pelvic organ prolapse. BJOG Int J Obstet Gynaecol.

[49] Devkota HR, Sijali TR, Harris C, Ghimire DJ, Prata N, Bates MN (2020). Bio-mechanical risk factors for uterine prolapse among women living in the hills of west Nepal: a case-control study. Womens Health.

